# Efficient, cell-based simulations of cardiac electrophysiology; The Kirchhoff Network Model (KNM)

**DOI:** 10.1038/s41540-023-00288-3

**Published:** 2023-06-14

**Authors:** Karoline Horgmo Jæger, Aslak Tveito

**Affiliations:** Simula Research Laboratory, Oslo, Norway

**Keywords:** Computer modelling, Biophysics, Differential equations, Computational biology and bioinformatics

## Abstract

Mathematical models based on homogenized representation of cardiac tissue have greatly improved our understanding of cardiac electrophysiology. However, these models are too coarse to investigate the dynamics at the level of the myocytes since the cells are not present in homogenized models. Recently, fine scale models have been proposed to allow for cell-level resolution of the dynamics, but these models are too computationally expensive to be used in applications like whole heart simulations of large animals. To address this issue, we propose a model that balances computational demands and physiological accuracy. The model is founded on Kirchhoff’s current law, and represents every myocyte in the tissue. This allows specific properties to be assigned to individual cardiomyocytes, and other cell types like fibroblasts can be added to the model in an accurate manner while keeping the computing efforts reasonable.

## Introduction

The healthy contraction of the cardiac muscle is initiated by an action potential (AP) generated in the sinoatrial node (SA-node). From the SA-node, the AP propagates as an electrochemical wave throughout the cardiac muscle approximately every second throughout an individual’s lifespan. The AP commences with a significant increase in the membrane potential of each cell, leading to an influx of calcium into the cell. This, in turn, triggers the release of large amounts of calcium from intracellular storage systems. The resultant rise in intracellular calcium concentration induces contraction of each myocyte, which underlies the heart’s essential pumping function.

Remarkably stable and versatile, this process automatically adapts to the body’s needs and generally operates for many years without external maintenance, exemplifying an incredible biological machinery. Nevertheless, the process can fail, causing extensive harm to the body and potentially leading to death. Gaining a solid understanding of cardiac function is critical for comprehending how to address related illnesses.

In many scientific fields, understanding is often grounded in mathematical models that can be employed to examine the dynamics of a particular system. Computational cardiology has advanced significantly since the groundbreaking paper by Hodgkin and Huxley^1^, which modeled excitable neurons, and the subsequent paper by Noble^2^ that introduced the first model of a cardiac AP. Today, knowledge of cardiac electrophysiology is to a large degree founded on computational models of the heart.

**Fig. 1:**
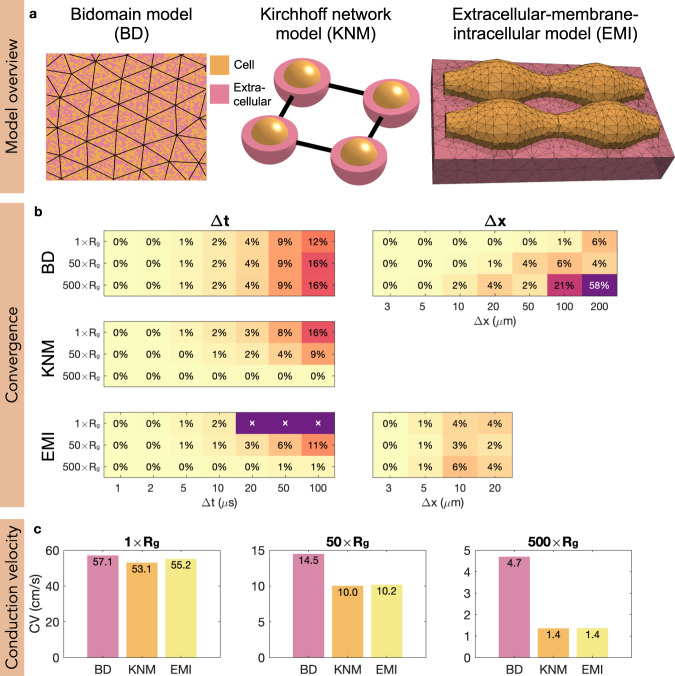
Model comparison and convergence. **a** Illustration of the models. In BD, the extracellular space, the membrane and the intracellular space are assumed to exist everywhere in the tissue, and the computational mesh can therefore be chosen irrespective of the location of the cells. In KNM, each cell and an associated part of the extracellular space are represented as computational nodes connected to their neighbors. In EMI, the computational nodes are set up to spatially resolve the extracellular space, the cell membrane, and the intracellular space. Note that in the illustration, the extracellular space only partly covers the cells to make the cells visible, but in simulations, it surrounds the cells on all sides. **b** Convergence analysis. The tables display the difference, in percent, between the CV computed using the finest considered resolution (Δ*t* = 1 μs, Δ*x* = 3 μm) and coarser resolutions for three different adjustments of the default gap junction resistance, *R*_*g*_. The discretization parameter that is not varied is fixed at the finest resolution. Note that for KNM, there is no spatial discretization since the computational nodes are the cells, and for EMI, the applied numerical scheme is unstable for 1 × *R*_*g*_ if Δ*t* ≥ 20 μs^29^. **c** Comparison of the CV for the three models. The CV is computed using the finest considered resolution for three choices of *R*_*g*_. Note the change of definition of the axes for the three panels.

The present state-of-the-art simulators of cardiac tissue are based on the homogenized bidomain model (BD)^3^. This model is accurate at the millimeter level^4^, and has been successfully applied to study a series of challenging questions in cardiac electrophysiology^5–7^. The bidomain model is very well established with well tested, stable and openly available software, see, e.g., https://opencarp.org/. However, since the myocyte is not present in the model, it is very difficult to use this approach to model cell-to-cell variations that appear during illness. Fundamentally, the bidomain model assumes that the extracellular space, the cell membrane and the intracellular space are present *everywhere* (see Fig. 1a). When the mesh resolution reaches the length scale of the myocyte, this approximation runs into difficulties because the cell is not part of the model.

Recently, a cell-based mathematical model has been suggested where the extracellular (E) space, the cell membrane (M) and the intracellular space (I) are explicitly represented as distinct and spatially resolved parts of the computational domain (see Fig. 1a). This model will herafter be referred to as the extracellular-membrane-intracellular (EMI) model^4,8,9^. The EMI model has been applied to study properties of cardiac conduction^10,11^, and arrhythmogenic influence of mutations in the sleeve of the pulmonary veins^12^. It has also been compared to the bidomain model^13–15^, and solved using a boundary integral formulation^16^. The main advantage of EMI is that the cell is represented in the model and the properties of each cell can therefore be adjusted and cell-level accuracy can be achieved. Furthermore, local changes due to illness can be properly analyzed. The main disadvantage, however, is that the EMI model is very CPU demanding.

The EMI model provides subcellular precision, as it enables manipulation of properties *within* individual myocytes^10,17^. Although this offers great flexibility, it is not required in all types of simulation. Here, we will present the Kirchhoff network model (KNM) which represents each myocyte and allows manipulation at the cellular level, and between cells, but not within individual cells. The Kirchhoff network model is based on representing each cell and its surrounding extracellular space as computational nodes and applying Kirchhoff’s current law, stating that the sum of currents into a node must equal the sum of currents out of the node. We will show that KNM is much less computationally demanding than the EMI model and has computational demands that are comparable to the bidomain model.

## Models

### The bidomain model (BD)

The bidomain model (see, e.g., ref. ^3^) is based on the assumption that the extracellular space, the membrane and the intracellular space all exist everywhere in the tissue. The model is expressed as a system of two partial differential equations (PDEs) for the unknown functions *v* and *u*_*e*_, representing the membrane potential and the extracellular potential, respectively (both in mV). This system is coupled to a system of ordinary differential equations for the membrane dynamics with an additional set of unknown state variables, *s*, representing ion channel gates and ionic concentrations. The system reads

1 $${C}_{m}\frac{\partial v}{\partial t}={\chi }^{-1}\left(\nabla \cdot \left({M}_{i}\nabla v\right)+\nabla \cdot \left({M}_{i}\nabla {u}_{e}\right)\right)-{I}_{{{{\rm{ion}}}}}(s,v),$$

2 $$0=\nabla \cdot \left({M}_{i}\nabla v\right)+\nabla \cdot \left(({M}_{i}+{M}_{e})\nabla {u}_{e}\right),$$

3 $$\frac{ds}{dt}=F(s,v).$$

Here, *C*_*m*_ is the specific membrane capacitance (in μF/cm^2^), *χ* is the membrane area to volume ratio (in cm^−1^), *M*_*i*_ and *M*_*e*_ are intracellular and extracellular bidomain conductivity tensors (in mS/cm), *I*_ion_ is the current density through in channels, pumps and exchangers in the cell membrane (in μA/cm^2^) and *F* is a function governing the dynamics of the state variables *s*.

### The Kirchhoff network model (KNM)

Here, we introduce the Kirchhoff network model based on representing each cell and an associated surrounding extracellular space as computational nodes and applying Kirchhoff’s current law.

We assume that currents of the form

4 $${I}_{i}^{j,k}={G}_{i}^{j,k}\left({u}_{i}^{j}-{u}_{i}^{k}\right),$$

5 $${I}_{e}^{j,k}={G}_{e}^{j,k}\left({u}_{e}^{j}-{u}_{e}^{k}\right)$$

flow between all connected neighboring cells *j* and *k* and neighboring extracellular compartments *j* and *k*, respectively. Here, ${G}_{i}^{j,k}$ and ${G}_{e}^{j,k}$ are the total intracellular and extracellular conductances (in mS), and ${u}_{i}^{k}$ and ${u}_{e}^{k}$ are the intracellular and extracellular potentials (in mV) in cell *k* and extracellular compartment *k*, respectively.

In addition, we assume that membrane currents of the form

6 $${I}_{m}^{k}={A}_{m}^{k}\left({C}_{m}\frac{d{v}^{k}}{dt}+{I}_{{{{\rm{ion}}}}}^{k}({v}^{k},{s}^{k})\right)$$

flow between a cell and its associated extracellular space. Here, ${A}_{m}^{k}$ is the membrane area of cell *k* (in cm^2^), *C*_*m*_ is the specific membrane capacitance (in μF/cm^2^), ${v}^{k}={u}_{i}^{k}-{u}_{e}^{k}$ is the membrane potential of cell *k* (in mV), ${I}_{{{{\rm{ion}}}}}^{k}$ is the ionic current density through ion channels, pumps and exchangers on the membrane of cell *k* (in μA/cm^2^) and *s*^*k*^ is a set of additional state variables modeling the membrane dynamics of cell *k*.

The sign of the current flowing across the cell membrane depends on the charge of the ions and the direction of flow. In the context of cardiac electrophysiology, it is common to focus on positive ions such as Na^+^, K^+^, and Ca^2+^. By convention, the flow of positive ions out of the cell is defined as positive. Therefore, the membrane current ${I}_{m}^{k}$ is positive when the ions under consideration flow from the intracellular space to the extracellular space. Similarly, ${I}_{i}^{j,k}$ is defined as positive when positive charges flow from cell *j* to cell *k*.

Taking the defined current directions into account, for each cell *k*, the membrane currents ${I}_{m}^{k}$ flow out of the cell into its associated extracellular space and the sum of currents ${\sum }_{j\in {N}_{k}}{I}_{i}^{j,k}$ flow into the cell from neigbouring cells. Here, *N*_*k*_ denotes the collection of connected neighboring cells of cell *k*. Applying Kirchhoff’s current law, stating that the sum of currents into a cell must equal the sum of currents out of the cell, we get

7 $${I}_{m}^{k}=\mathop{\sum}\limits_{j\in {N}_{k}}{I}_{i}^{j,k}.$$

Similarly, by applying Kirchhoff’s current law to the extracellular space surrounding cell *k*, we get

8 $${I}_{m}^{k}+\mathop{\sum}\limits_{j\in {N}_{k}}{I}_{e}^{j,k}=0.$$

Inserting (7) in (8), we get

9 $$\mathop{\sum}\limits_{j\in {N}_{k}}{I}_{i}^{j,k}+\mathop{\sum}\limits_{j\in {N}_{k}}{I}_{e}^{j,k}=0,$$

which means that we end up with the system

10 $${C}_{m}\frac{d{v}^{k}}{dt}=\frac{1}{{A}_{m}^{k}}\mathop{\sum}\limits_{j\in {N}_{k}}{I}_{i}^{j,k}-{I}_{{{{\rm{ion}}}}}^{k}({v}^{k},{s}^{k}),$$

11 $$0=\mathop{\sum}\limits_{j\in {N}_{k}}{I}_{i}^{j,k}+\mathop{\sum}\limits_{j\in {N}_{k}}{I}_{e}^{j,k},$$

12 $$\frac{d{s}^{k}}{dt}={F}_{k}({s}^{k},{v}^{k}),$$

where we have included the system of equations for the additional state variables, *s*^*k*^, involved in the definition of ${I}_{{{{\rm{ion}}}}}^{k}$. In order to express the first two equations in terms of just two variables, $v^k$ and ${u}_{e}^{k}$ instead of the three variables ${v}^{k},{u}_{i}^{k}$ and ${u}_{e}^{k}$, we can use the definition of ${v}^{k}={u}_{i}^{k}-{u}_{e}^{k}$ to replace ${u}_{i}^{k}$ in the currents of the form (4) by ${v}^{k}+{u}_{e}^{k}$.

### Spatial definition of KNM

In KNM, the network’s spatial configuration is determined by the center of mass of each myocyte. Assuming uniform myocyte size and distribution, the network takes the form of a regular, uniform mesh. This implies that the cellular structure inherently defines the network, eliminating the need for any additional spatial mesh parameters aside from the size and position of individual cells. Consequently, mesh refinement or coarsening is not applicable to KNM. The computational load can be modified by changing the total number of cells, but the spatial resolution remains defined by the cells.

### The extracellular-membrane-intracellular model (EMI)

In the EMI model (see, e.g., refs. ^8,9^), the extracellular space, Ω_*e*_, and the intracellular spaces of the cells, ${\Omega }_{i}^{k}$, are represented as spatially resolved volumes. The cell membrane, Γ_*k*_, is defined at the interface between ${\Omega }_{i}^{k}$ and Ω_*e*_. Furthermore, intercalated discs, Γ^*k*,*j*^ are defined at the interface between two neighboring cells, ${\Omega }_{i}^{k}$ and ${\Omega }_{i}^{j}$. The unknown functions *u*_*e*_ and ${u}_{i}^{k}$ representing the extracellular and intracellular potentials (in mV) are only defined in Ω_*e*_ and ${\Omega }_{i}^{k}$, respectively. Similarly, the membrane potential, ${v}^{k}={u}_{i}^{k}-{u}_{e}^{k}$, and the additional state variables of the membrane model, *s*^*k*^, are defined only at the membrane Γ_*k*_, and the intercalated disc potentials, ${w}_{k}={u}_{i}^{k}-{u}_{i}^{j}$, are defined at the intercalated discs Γ^*k*,*j*^ of cell *k*. The system of equations reads

13 $$\nabla \cdot {\sigma }_{i}\nabla {u}_{i}^{k}=0\quad \,{{\mbox{in}}}\,\,{\Omega }_{i}^{k},$$

14 $$\nabla \cdot {\sigma }_{e}\nabla {u}_{e}=0\quad \,{{\mbox{in}}}\,\,{\Omega }_{e},$$

15 $${C}_{m}\frac{\partial {v}^{k}}{\partial t}+{I}_{{{{\rm{ion}}}}}^{k}({v}^{k},{s}^{k})={n}_{e}\cdot {\sigma }_{e}\nabla {u}_{e}=-{n}_{i}^{k}\cdot {\sigma }_{i}\nabla {u}_{i}^{k}\quad \,{{\mbox{at}}}\,\,{\Gamma }_{k},$$

16 $$\frac{\partial {s}^{k}}{\partial t}={F}_{k}({s}^{k},{v}^{k})\quad \,{{\mbox{at}}}\,\,{\Gamma }_{k},$$

17 $${C}_{g}\frac{\partial {w}^{k}}{\partial t}+{I}_{{{{\rm{gap}}}}}^{k,j}({w}^{k})={n}_{i}^{j}\cdot {\sigma }_{i}\nabla {u}_{i}^{j}=-{n}_{i}^{k}\cdot {\sigma }_{i}\nabla {u}_{i}^{k}\quad \,{{\mbox{at}}}\,\,{\Gamma }_{k,j},$$

for all cells *k* and all *j* ∈ *N*_*k*_, where *N*_*k*_ defines all the neighbors of cell *k*. Here, *σ*_*i*_ and *σ*_*e*_ (in mS/cm) are the conductivities of the intracellular and extracellular spaces, respectively, *C*_*m*_ and *C*_*g*_ are the specific capacitance (in μF/cm^2^) of the membrane and the intercalated discs, respectively, ${I}_{{{{\rm{ion}}}}}^{k}$ is the ionic current density (in μA/cm^2^) through ion channels, pumps and exchangers in the membrane of cell $k,{n}_{i}^{k}$ and *n*_*e*_ are the outward pointing unit normal vectors of ${\Omega }_{i}^{k}$ and Ω_*e*_, respectively, and *F*_*k*_ governs the dynamics of the additional membrane state variabels, *s*^*k*^. Moreover, ${I}_{{{{\rm{gap}}}}}^{k,j}$ is the current density through gap junctions connecting cells *k* and *j* given by the passive model

18 $${I}_{{{{\rm{gap}}}}}^{k,j}({w}^{k})=\frac{1}{{R}_{g}^{k,j}}{w}^{k},$$

where ${R}_{g}^{k,j}$ is the gap junction resistance density (in kΩ cm^2^) of the intercalated disc connecting cells *k* and *j*.

## Results

One crucial feature of the electrochemical wave that triggers the contraction of the heart muscle is its conduction velocity (CV). It is well known that reduced CV can cause arrhythmias by enabling reentry of the wave in a large heart volume, see, e.g., ref. ^18^. Therefore, accurate computation of the CV is essential. To compare the results of BD, KNM and EMI, we first determine the appropriate time step and mesh parameters ensuring that the numerical solutions are converged. Next, we investigate how the three models predict different CVs under conditions where conduction is impaired between cells. In our example, the reduction of the CV is implemented by gradual increase of the parameter *R*_*g*_, representing cell-to-cell resistance. Physiologically, increased resistance can, for instance, be caused by fibrosis, see, e.g., ref. ^19^. The cell-to-cell resistance, *R*_*g*_, enters BD as shown in Eqs. (66)–(68) in ref. ^14^. For KNM, *R*_*g*_ is included as shown in Eq. (20) in the ’Methods’ section, and for EMI *R*_*g*_ is included as shown in Eq. (18).

After observing model differences in CV response to an increased gap junction resistance, we demonstrate that these differences lead to different predictions of reentry, which we initiate using an S1–S2 stimulus protocol. In this protocol, S1 represents the primary stimulus of cardiac tissue, and S2 represents a premature, ectopic beat, see, e.g., refs. ^20,21^. Here, we adopt the S1–S2 protocol from ref. ^4^. It should be noted that we use CV to assess the accuracy of the models because of its physiological relevance and importance. However, other mathematical norms may prove more sensitive to changes in physiological properties, see, e.g., ref. ^22^.

### Convergence of numerical solutions

Our aim is to compare the physiological accuracy and computational demands of BD, KNM and EMI, and we start by investigating the required resolutions. Figure 1b displays the difference, in percent, between the conduction velocity (CV) computed using the finest considered resolution and a number of coarser resolutions for each model. We consider three different choices of gap junction resistance between the cardiomyocytes; normal conduction (1 × *R*_*g*_), reduced conduction (50 × *R*_*g*_) and severely reduced conduction (500 × *R*_*g*_). Assuming that we want the difference to the finest resolution to be 2% or below, we observe that a temporal resolution of 10 μs appears to be sufficient for all models. Similarly, for BD and EMI we need a 10 μm and a 5 μm spatial resolution, respectively. For KNM, there is no adjustable spatial discretization because the location of the cells defines the node points.

### Comparison of model solutions

**Fig. 2:**
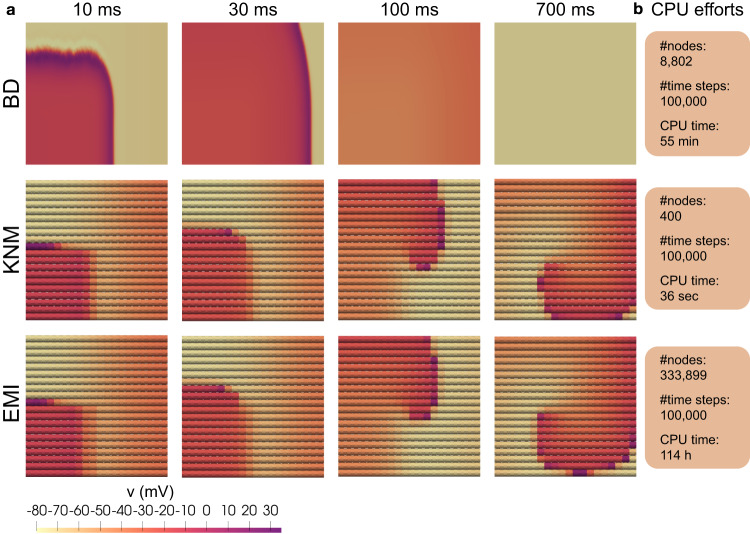
Micro-reentry and CPU efforts. **a** A spiral wave is generated for KNM and EMI, but not for BD. We consider a collection of 20 × 20 weakly coupled cells (500 × *R*_*g*_) and apply an S2 stimulation (see, e.g., refs. ^20,21^) in the lower left quarter of the domain 240 ms after an S1 stimulation was applied in the left part of the domain. Note that also other tested timings of the S2 stimulation did not give reentry for BD (see Supplementary Figure S1). The time points displayed at the top of the plots report the time (after the S2 stimulation) when the snapshots are recorded. Note that the longitudinal axis is scaled for improved visibility. **b** CPU efforts associated with the simulations. We report the number of mesh nodes, the number of time steps and the CPU time required to run the simulation. The applied resolution is Δ*t* = 10 μs (all models), Δ*x* = 10 μm (BD), and Δ*x* = 5 μm (EMI).

Next, we investigate the accuracy of BD and KNM by comparing their solution to the solution of the more detailed EMI model. The conduction velocities computed for each model are reported in Fig. 1c. We observe that for normal conduction conditions, the models all display similar conduction velocities, but when the conduction is impaired, there is a significant difference between BD and the two other models. This indicates that both BD and KNM are accurate in normal conduction conditions, but that KNM is considerably more accurate in cases of reduced cell coupling. An example where the inaccuracy of BD has important consequences is illustrated in Fig. 2a. Using an S1-S2 stimulation protocol (see, e.g., refs. ^4,20,21^) in a collection of weakly coupled cells, a reentrant spiral wave is generated for KNM and EMI, but for BD, the CV is too high for a reentrant spiral wave to be generated.

### Computational demands

In Fig. 2b, we report the CPU efforts for the simulations displayed in Fig. 2a. We observe that KNM, in addition to providing very similar results to EMI, is by far the least computationally demanding model, with a CPU time of ~1% and 0.01% of BD and EMI, respectively. Based on the results presented in Fig. 1, we concluded that a spatial resolution of 10 μm was necessary to ensure that the error of the CV is less than 2% for BD. This is in contrast to earlier studies where spatial resolutions of about Δ*x* ≈ 250 μm have been applied^23,24^. Whereas the spatial mesh of KNM is completely determined by the location of the individual cardiomyocytes, the necessary resolution of BD is dictated by the required accuracy of the numerical solution. The comparison of CPU demands between BD and KNM thus depends on the accuracy demanded by the specific application under consideration. In general, the CPU demands of KNM and BD appear to be comparable, but both are clearly faster than EMI.

### KNM as a discrete version of BD

Upon inspection of the equations defining BD, we observe that discretizing them on a standard finite difference lattice with the cell length as the spatial discretization parameter results in a numerical model that closely resembles KNM. Thus, KNM can be interpreted as a special case of the discrete BD. However, as noted in Fig. 1, KNM does not represent the converged solution of BD.

## Discussion

The bidomain model (BD) is widely regarded as the state-of-the-art model for numerical simulation of cardiac electrophysiology. The model has acceptable computational demands, but lacks accuracy at the level of individual cardiomyocytes. The EMI model is a recently established cell-based alternative to BD with subcellular accuracy at the expense of very high computational demands. Here, we have presented the KNM approach that achieves cell-level accuracy at reasonable computational demands. Specifically, Fig. 2 shows that KNM requires considerably less computing efforts than EMI while maintaining the conduction properties of the detailed EMI model in the examples considered here (see Figs. 1 and 2). It should, however, be noted that KNM cannot reach the accuracy of EMI since EMI offers sub-cellular accuracy. The computational complexity of KNM is intuitively easy to comprehend, as it is solely based on the representation of every cell in the tissue. It should be noted that the KNM approach shares similarities with earlier models^21,25,26^. In the present report, KNM is directly grounded in the biophysics of cell interactions with neighboring cells and with the extracellular domain. This implies that the model works equally well in 1D, 2D and 3D, and that other cell types (e.g., fibroblasts) can be added to the model in a straightforward manner. Excitable tissue is found in many organs and the KNM approach can in principle always be applied to model such collections of cells. However, we only have experience in applying it to collections of cardiomyocytes.

## Methods

### Membrane model

In our simulations, we let the membrane dynamics defining *I*_ion_ and *F* be governed by the left atrial membrane model defined in ref. ^12^.

### Parameters

The specific model parameters used in our simulations are *σ*_*i*_ = 4 mS/cm, *σ*_*e*_ = 20 mS/cm, *C*_*m*_ = 1 μF/cm^2^, *C*_*g*_ = 0.5 μF/cm^2^, and *R*_*g*_ = 0.0015 kΩ cm^2^. This specified value of *R*_*g*_ represents the default case of normal conduction. The cardiomyocytes are 120 μm long and have a radius varying from 6 *μ*m at the cell ends to 7 μm at the center of the cell. In EMI, we let the distance from the boundary of the extracellular space to the cell collection be 2 μm in all spatial directions.

The paper ref. ^14^ provides formulas used to define the parameters *χ*, *M*_*i*_ and *M*_*e*_ of the bidomain model from the EMI model parameters and mesh. Similar formulas for KNM are based on classical arguments of electrical conductance (see, e.g., ref. ^27^) and given by

19 $${G}_{e}^{j,k}={\delta }_{e}^{j,k}\frac{{A}_{j,k}{\sigma }_{e}}{{l}_{j,k}},$$

20 $${G}_{i}^{j,k}=\frac{1}{\frac{{l}_{j,k}}{{\delta }_{i}^{j,k}{A}_{j,k}{\sigma }_{i}}+\frac{{R}_{g}}{{A}_{g}^{j,k}}},$$

where *A*_*j*,*k*_ is the average cross sectional area between compartments *j* and *k* (including both the intracellular and extracellular parts), and *l*_*j*,*k*_ is the distance between the cell centers. For example, for two cells connected along the *x*-axis, we typically have *A*_*j*,*k*_ = *l*_*z*_*l*_*y*_ and *l*_*j*,*k*_ = *l*_*x*_, where *l*_*x*_, *l*_*y*_ and *l*_*z*_ are the lengths in the *x*-, *y*-, and *z*-directions, respectively, of each compartment containing a cell and an associated extracellular volume. Furthermore, ${\delta }_{e}^{j,k}$ and ${\delta }_{i}^{j,k}$ are the extracellular and intracellular fractions, respectively, of the volume between the centers of cell *j* and *k*. In our simulations, these fractions do not vary across the domain and can be computed from the EMI model mesh by

21 $${\delta }_{e}^{j,k}=\frac{{\int}_{{\Omega }_{e}}1\,dV}{{\int}_{\Omega }1\,dV},\quad {\delta }_{i}^{j,k}=\frac{{\int}_{{\Omega }_{i}}1\,dV}{{\int}_{\Omega }1\,dV},$$

where Ω_*e*_ and Ω_*i*_ are the extracellular and intracellular parts of the domain, respectively, and Ω denotes the entire domain. Similarly, ${A}_{g}^{j,k}$ is the area of the intercalated disc connecting cell *j* and *k*, and ${A}_{m}^{k}$ (see (10)) is the membrane area of a cell, which both can be computed from the EMI model mesh.

### Boundary conditions

For the simulations reported in Fig. 1, we consider a 1D strand of 15 cells. For BD and KNM, this results in a 1D problem and we apply a homogeneous Dirichlet boundary condition for *u*_*e*_ and homogeneous Neumann boundary conditions for *u*_*i*_ at the two (left and right) boundaries of the 1D strand. For the EMI model, the 1D strand of cells is represented in 3D with homogeneous Dirichlet boundary conditions on the leftmost and rightmost extracellular boundaries and homogeneous Neumann boundary conditions on the remaining outer extracellular boundaries.

For the simulations reported in Fig. 2, we consider a collection of 20 × 20 cells. For BD and KNM, this results in a 2D problem, and we apply the same boundary conditions as those described above for the entire domain boundary. For the EMI model, the problem is still 3D and we extend the homogeneous Dirichlet boundary conditions to the outer extracellular boundary in both the *x*- and *y*-directions to make the problem equivalent to that defined for BD and KNM.

### Stimulus current

For the simulations reported in Fig. 1, we apply a stimulus current to the membrane model in a part of the domain corresponding to the location of the leftmost cell. For BD, this corresponds to *x* ∈ [0, 120 μm], for KNM, this corresponds to node number 1, and for EMI this corresponds to *x* ∈ [2 μm, 122 μm] and all values of *y* and *z*. For the simulations reported in Fig. 2, we apply a first (S1) stimulus current to a part of the domain corresponding to the leftmost row of cells. For BD, this corresponds to *x* ∈ [0, 120 μm] and all values of *y*, for KNM, this corresponds to the row of leftmost nodes, and for EMI this corresponds to *x* ∈ [2 μm, 122 μm] and all values of *y* and *z*. Then, after 240 ms, we apply a second (S2) stimulus current to the lower left quarter of the domain. The applied stimulus current has a strength of 40 μA/cm^2^ and lasts for 2 ms for both Figs. 1 and 2 and for both the S1 and S2 stimulations.

### Definition of conduction velocity

The conduction velocities reported in Fig. 1 are computed by recording the difference between the points in time when the membrane potential in the two points corresponding to the centers of cell numbers 3 and 13 reach a value ≥−20 mV. More specifically, the conduction velocity is defined as the distance between these two points divided by the difference in time.

### Numerical methods

We apply a classical first-order temporal operator splitting scheme to split the solution of the linear and non-linear parts of BD and KNM^28^. Similarly, the EMI model is solved using the spatial and temporal operator splitting technique from^29^. The PDEs of BD and EMI are solved using the MFEM finite element software^30^ and the meshes are generated using gmsh^31^. The linear part of the KNM system is solved using a standard implicit (backward Euler) scheme. The linear systems of all the models are solved using the generalized minimal residual method, except for the intracellular systems of the EMI model, which are solved using the conjugate gradient method. The non-linear system of ODEs describing the membrane dynamics is solved using a first-order Rush-Larsen scheme^32,33^ generated using the Gotran code generator^34^ and OpenMP parallelization^35^. All simulations are run using C++.

### Reporting summary

Further information on research design is available in the Nature Research Reporting Summary linked to this article.

## Supplementary information

Supplementary Information

Reporting Summary

## Data Availability

The data created in this study are available at 10.5281/zenodo.7848664^36^.

## Code availability

The software used to perform the numerical simulations in this study is available at 10.5281/zenodo.7848664^36^. Running the code requires the MFEM C++ library for finite element methods available at https://mfem.org/download/. MFEM version 4.1 was used in this study. In addition, ParaView (version 5.11.0), available at https://www.paraview.org/download/ was used to create the visualizations in Fig. 2.
