# Supplementary material for: Efficient, cell-based simulations of cardiac electrophysiology; The Kirchhoff Network Model (KNM)
Source: NPJ Syst Biol Appl. 2023 Jun 14;9:25. doi: 10.1038/s41540-023-00288-3 (PMC10267147; doi:10.1038/s41540-023-00288-3)
Supplement: Supplementary file 1 — Supplementary Information [file 41540_2023_288_MOESM1_ESM.pdf]

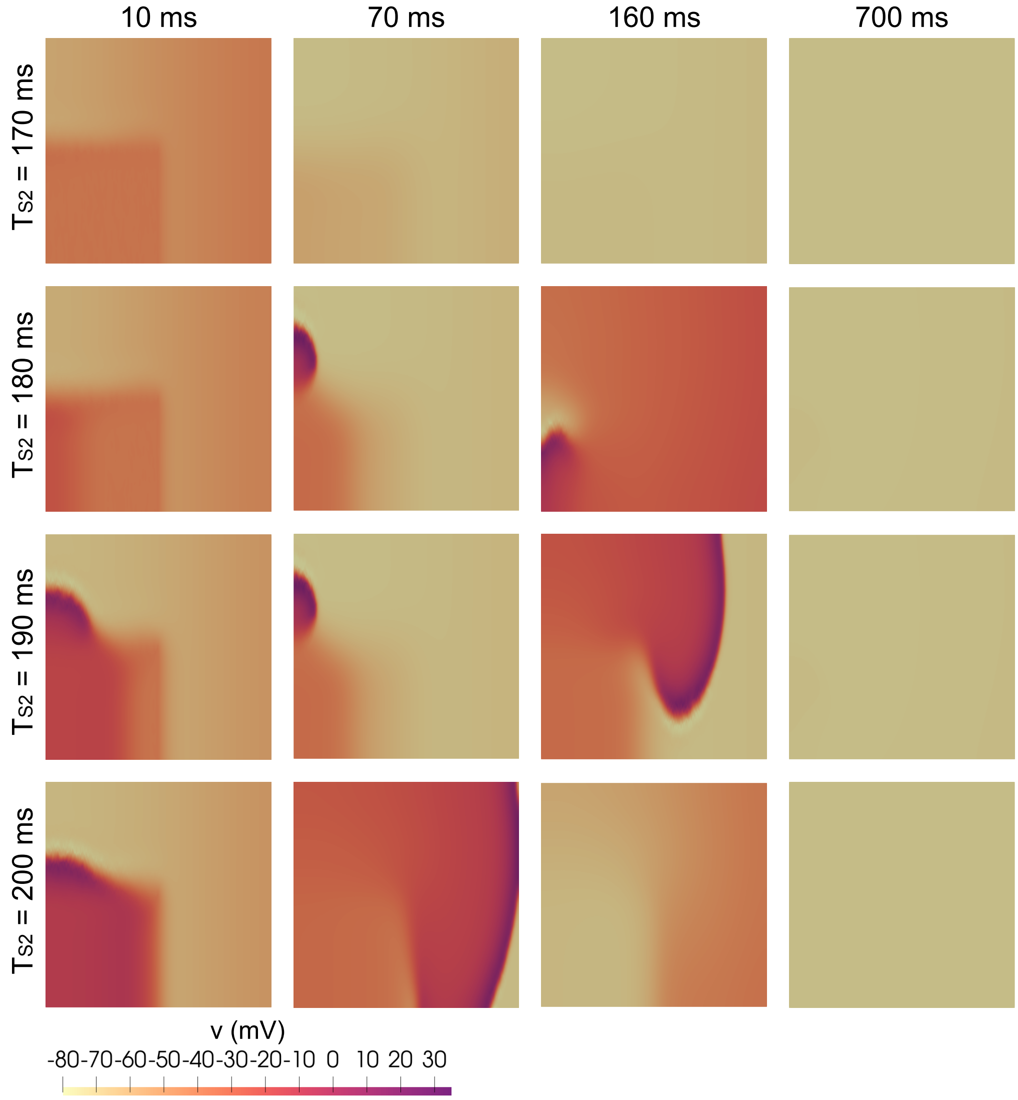

Supplementary Figure 1: Attempts to generate a spiral wave for the bidomain model by varying the timing of the S2 stimulation. Each figure row represents a specific timing of the S2 simulation, T_{S2}, after the S1 stimulation. This S2 timing is reported to the left of the snapshots. The time points displayed above the snapshots report the time when the snapshots are recorded after the S2 stimulation was applied. We observe that none of the considered S2 timings result in a reentrant spiral wave. Note that, as in Figure 2 in the paper, the x-axis of the snapshots has been adjusted to get a square shape.
